# Association between *BCL11B* gene polymorphisms and age-related hearing loss in the elderly: A case-control study in Qingdao, China

**DOI:** 10.1371/journal.pone.0304770

**Published:** 2024-06-03

**Authors:** Xin Li, Jingkai Zhang, Hua Zhang, Jifeng Ren, Hainan Cao, Yaoyao Xu, Dongfeng Zhang, Haiping Duan

**Affiliations:** 1 Department of Epidemiology and Health Statistics, Public Health College, Qingdao University, Laoshan District, Qingdao, Shandong, China; 2 Qingdao Municipal Center for Disease Control and Prevention, Shibei District, Qingdao, Shandong, China; 3 Shandong Provincial Chronic Disease Hospital, Shinan District, Qingdao, Shandong, China; 4 Department of Otorhinolaryngology, Qingdao Municipal Hospital, Shinan District, Qingdao, Shandong, China; 5 School of Public Health, Weifang Medical University, Weicheng District, Weifang, Shandong, China; Aichi Prefectural Mikawa Aoitori Medical and Rehabilitation Center for Developmental Disabilities, JAPAN

## Abstract

Age-related hearing loss is a complex disease caused by a combination of genetic and environmental factors, and a study have conducted animal experiments to explore the association between *BCL11B* heterozygosity and age-related hearing loss. The present study used established genetic models to examine the association between *BCL11B* gene polymorphisms and age-related hearing loss. A total of 410 older adults from two communities in Qingdao, China, participated in this study. The case group comprised individuals aged ≥ 60 years with age-related hearing loss, and the control group comprised individuals without age-related hearing loss from the same communities. The groups were matched 1:1 for age and sex. The individual characteristics of the participants were analyzed descriptively using the Mann–Whitney U test and the chi-square test. To explore the association between *BCL11B* gene polymorphisms and age-related hearing loss, conditional logistic regression was performed to construct genetic models for two single-nucleotide-polymorphisms (SNPs) of *BCL11B*, and haplotype analysis was conducted to construct their haplotype domains. Two SNP sites of the *BCL11B* gene, four genetic models of rs1152781 (additive, dominant, recessive, and codominant), and five genetic models of rs1152783 (additive, dominant, recessive, codominant, and over dominant) were significantly associated with age-related hearing loss in the models both unadjusted and adjusted for all covariates (*P* < 0.05). Additionally, a linkage disequilibrium between rs1152781 and rs1152783 was revealed through haplotype analysis. Our study revealed that *BCL11B* gene polymorphisms were significantly associated with age-related hearing loss.

## Introduction

Age-related hearing loss (ARHL), also known as presbycusis, is a complex disease characterized by bilateral and symmetrical sensorineural hearing loss that progresses with age because of the cumulative effects of aging on the auditory system [1–3]. The World Report on Hearing 2021 estimated that approximately 2.5 billion people will experience hearing loss by the year 2050 [4]. With the population aging, more than half of the middle-aged and older adults in China are experiencing various degrees of hearing loss [5,6]. Hearing loss has an adverse effect on the quality of life of older adults, and it can also affect their gait stability [7], driving ability [8], and functional decline [9].

ARHL is a complex disease caused by a combination of genetic and environmental factors, the potential risk factors for ARHL include biological age, sex, ethnicity, lifestyle factors (e.g., smoking, drinking, diet), comorbidities (e.g., hypertension, diabetes, atherosclerosis), environmental factors (e.g., noise exposure, ototoxic medications), and genetic predisposition [10]. A Chinese study based on middle-aged and elderly twin groups has reported that the heritability of pure-tone average (PTA) at middle and high frequencies was 34.77% and 43.26%, respectively [6]. Numerous studies have used humans and animal models to study various genes, including *GRM7*, *NR3C1*, *CDH23*, *HSPA1A*, *HSPA1L*, and *HSPA1B* [2,11–13], all of which have been reported to be associated with ARHL.

Previous studies have revealed that *BCL11B* is involved in the differentiation, proliferation and survival of multiple cell types [14–16]. *BCL11B* was also discovered to be expressed in mouse cochlear cells [17]. In a study that compared mice that were knocked out *BCL11B* gene with wild-type mice, showing that the absence of *BCL11B* expression resulted in the loss of outer hair cells and an increased auditory brainstem response (ABR) threshold in mice, suggesting that the expression of *BCL11B* is associated with ARHL [17]. However, no study has clarified the association of *BCL11B* with ARHL in human population.

To the best of our knowledge, this study is the first to explore the association between *BCL11B* gene polymorphisms and ARHL susceptibility in human population. It is hoped that the results of this study will provide a basis for future researches regarding ARHL and contribute to the screening and treatment schedules for ARHL in the future.

## Materials and methods

### Study participants

The participants of this study comprised older adults from two communities in Qingdao, China. Nonrelated ethnic Han Chinese individuals aged ≥ 60 years who had ARHL were recruited to form the case group on the basis of specific inclusion and exclusion criteria. From the same community, nonrelated ethnic Han Chinese individuals without ARHL, who were matched 1:1 with those in the case group for age and sex, were recruited to form the control group. In total, we recruited 410 individuals within the period of December 31, 2018 to June 7, 2019, of whom 205 were in the case group and 205 were in the control group. The investigators involved in the present study underwent training based on a standardized method before conducting a face-to-face survey with each participant; during each survey, a standard questionnaire was administered and completed. Our study was approved by the Medical Ethics Committee of the Qingdao Municipal Center for Disease Control and Prevention in Qingdao, China (ID:201804, approval data: July 12, 2018). All the participants signed an informed consent form to agree to participate in this study.

The inclusion criteria were as follows: (1) age ≥ 60 years or older; (2) non-related permanent Han Chinese residents in Qingdao (> 5 years); (3) fluent in communication and able to cooperate well with all survey projects; (4) voluntary participation.

The exclusion criteria were as follows: (1) a history of diseases affecting hearing, noise exposure and ototoxic drugs (e.g., congenital deafness, septic disease of the ear, continuous exposure to strong noise, streptomycin and other drugs taken and causing hearing damage); (2) those who were unable to cooperate autonomously in completing questionnaires and various tests.

### Audiometric examination

The external ear canals of the participants were examined and cleaned to ensure that they had normal structure and function. Pure-tone audiometry was conducted in the audiometry room by investigators who had undergone standardized training; an air-conduction pure-tone audiometer (Orbiter 922, Madsen) and special hearing test headphones (TDH39) were used to conduct hearing tests on the participants. Starting with the ear that each participant self-reported as having better hearing, audiometric measurements were performed at frequencies of 0.5, 1.0, 2.0, and 4.0 kHz for the left and right ears, respectively, with a reading accuracy of 1 dB.

In accordance with the criteria recommended by the World Health Organization (WHO) (1997), we calculated the participants′ PTA at frequencies of 0.5, 1.0, 2.0, and 4.0 kHz, and the PTA results for the better-hearing ear were then used for subsequent statistical analysis [18–20] because the better-hearing ear is typically the primary ear used by people in their daily lives [21]. On the basis of the aforementioned WHO criteria, the PTA of > 25 and ≤ 25 dB were applied to the case group and control group, respectively.

### Genotyping and quality controls

Two SNPs (rs1152781, rs1152783) of *BCL11B* were selected based on the following criteria: (1) functional SNPs in the promoter, 5′-untranslated region (5′-UTR), exon, and 3′-untranslated region (3′-UTR) of the *BCL11B* gene were identified from the National Center for Biotechnology Information SNP database NCBI-SNP (http://www.ncbi.nlm.nih.gov/snp/) and the 1000 Genomes East Asian database (https://www.ncbi.nlm.nih.gov/variation/tools/1000genomes/). The SNPs had to meet the requirements of the database regarding Han Chinese people in Beijing; specifically, their minimum allele frequency (MAF) needed to be > 0.05, and their minimum linkage disequilibrium correlation (r^2^) needed to be > 0.8. (2) SNPs associated with disease susceptibility were reported in the published literature [22,23]. Table 1 provides specific information about SNPs. Blood samples were taken, centrifuged, and separated within two hours. The DNA was extracted from the blood samples using the DNA extraction kit (available from BioTeke Corporation), and the quality of DNA was assessed using the NanoDrop 2000 spectrophotometer. Genotyping was carried out using the MassARRAY System, after PCR amplification, product alkaline phosphatase treatment, single-base elongation reaction, resin purification, then the resultant multiplex analyte mixture is transferred to a SpectroCHIP Array using a purpose-built dispenser (Agena Bioscience RS1000 Nanodispenser) and reactions were performed in 384-well SpectroCHIP Array, SpectroCHIP was analyzed using matrix-assisted laser desorption ionization time of flight (MALDI-TOF) mass spectrometry to finally obtain genotyping data and map[24]. With reference to the East Asian Samples of the 1000 Genomes Project, higher frequency alleles were used as the major alleles and lower frequency genes were used as the minor alleles.

**Table 1:** Basic information of SNPs.

Gene	SNP	Chromosome	Position (GRCh38.p14)	Type of mutation	Allele (major/minor)
*BCL11B*	rs1152781	14	99172933	3′ UTR	T/G
	rs1152783	14	99176023	Exon-synonymous	C/G

### Statistical analysis

For the data collected, the normality of continuous variables was first tested using the Kolmogorov-Smirnov normality test, means ± standard deviations were used to describe the normally distributed variables, and medians (interquartile ranges) were used to describe nonnormally distributed variables. For the normally distributed variables, two independent samples t-tests were performed to compare the means of the case and control groups. For the nonnormally distributed variables, the Mann-Whitney U test was performed. The chi-square test was conducted to compare differences in the distribution of categorical variables between two groups.

Each SNP was grouped into three categorical variables, with the wild type serving as the reference group. The Hardy-Weinberg equilibrium (HWE) of each SNP was then determined.

Using the chi-square test, the case and control groups were compared with respect to the genotype and allele distributions of the two SNPs of the *BCL11B* gene.

The identified associations between *BCL11B* gene polymorphisms and ARHL were analyzed using conditional logistic regression analysis by calculating odds ratios (ORs) and 95% confidence Intervals (95% CIs) for five genetic models (i.e., additive, dominant, recessive, co-dominant and over dominant) of the rs1152781 and rs1152783 of the *BCL11B* gene; in this step, two models were used, namely a model unadjusted for any covariates (model 1) and a model adjusted for all covariates (model 2). A two-sided *P* value of < 0.05 was regarded as statistically significant. We also performed haplotype analysis.

Haplotype analysis was performed by Haploview 4.1, and all other statistical analyses were conducted using IBM SPSS Statistics for Windows, version 26.0 (IBM, Armonk, NY, USA).

## Results

A total of 410 participants were included in the present study. In the case group, there were 205 participants, including 56 (27.32%) males and 149 (72.68%) females. In the control group, there were 205 participants, including 56 (27.32%) males and 149 (72.68%) females. The median age of both the case and control group was 65 (62, 68) years. Except for the PTA (*P* < 0.01), no statistical differences were identified between the case and control groups for demographic characteristics (Table 2).

**Table 2:** Demographics of the participants.

Variables	Control group	Case group	*P*
PTA (dB HL)^1^	21.25 (5.00)	32.50 (10.00)	**<0.001**
Smoking^2^			0.813
No	158 (77.07)	47 (22.93)	
Yes	160 (78.05)	45 (21.95)	
Drinking^2^			0.115
No	145 (70.73)	60 (29.27)	
Yes	130 (63.41)	75 (36.59)	
Diabetes^2^			0.899
No	167 (81.46)	38 (18.54)	
Yes	166 (80.98)	39 (19.02)	
Hypertension^2^			1.000
No	132 (64.39)	73 (35.61)	
Yes	132 (64.39)	73 (35.61)	
Cardiovascular disease^2^			1.000
No	107 (52.20)	98 (47.80)	
Yes	107 (52.20)	98 (47.80)	

^1^ Data are presented as median (interquartile range) and Mann-Whitney U test.

^2^ Data are presented as N (%) and Person’s chi-square test.

Further analysis showed that the distribution frequency of polymorphisms in *BCL11B* gene was consistent with the Hardy-Weinberg genetic equilibrium (*P* > 0.05). The genotypes of rs1152781 and rs1152783 were significantly different in both the case and control groups (*P* < 0.01) (Table 3). In addition, the distributions of the alleles of rs1152781 and rs1152783 were significantly different in both the case and control groups (*P* < 0.01) (Table 3).

**Table 3:** The distribution of genotype and allele of *BCL11B* compared between control group and case group.

SNP	Control group	Case group	*P*
rs1152781			
TT	142 (69.27)	109 (53.17)	**<0.001**
GT	58 (28.29)	82 (40.00)	
GG	2 (0.98)	14 (6.83)	
T	342 (84.65)	300 (73.17)	**<0.001**
G	62 (15.35)	110 (26.83)	
rs1152783			
CC	101 (49.27)	58 (28.29)	**<0.001**
CG	80 (39.02)	114 (55.61)	
GG	17 (8.29)	33 (16.10)	
C	282 (71.21)	230 (56.10)	**<0.001**
G	114 (28.79)	180 (43.90)	

The univariate conditional logistic regression analysis for rs1152781 revealed that all five genetic models were statistically significantly associated with ARHL. The multivariate conditional logistic regression analysis for rs1152781 revealed that four of the five genetic models were significantly associated with ARHL, with only the results for the over dominant model being nonsignificant (Table 4).

**Table 4:** Genetic models of the SNPs of the *BCL11B* gene.

SNP	Genetic model		Control group	Case group	OR^1^ (95%CI)	*P*	OR^2^ (95%CI)	*P*
rs1152781	Additive	TT vs GT vs GG	202	205	2.052 (1.419, 2.969)	**<0.001**	2.418 (1.492, 3.918)	**<0.001**
	Dominant	TT	142	109				
		GT+GG	60	96	2.029 (1.346, 3.060)	**0.001**	2.285 (1.343, 3.888)	**0.002**
	Recessive	TT+GT	200	191				
		GG	2	14	7.000 (1.591, 30.800)	**0.010**	23.304 (2.311, 215.228)	**0.007**
	Co-dominant	TT	142	109				
		GT	58	82	1.800 (1.180, 2.745)	**0.006**	1.860 (1.064, 3.249)	**0.029**
		GG	2	14	8.655 (1.934, 38.734)	**0.005**	27.081 (2.781, 263.749)	**0.005**
	Over dominant	TT+GG	144	123				
		GT	58	82	1.622 (1.077, 2.443)	**0.021**	1.640 (0.961, 2.797)	0.070
rs1152783	Additive	CC vs CG vs GG	198	205	1.982 (1.435, 2.738)	**<0.001**	2.230 (1.504, 3.307)	**<0.001**
	Dominant	CC	101	58				
		CG+GG	97	147	2.406 (1.593, 3.634)	**<0.001**	2.893 (1.733, 4.829)	**<0.001**
	Recessive	CC+CG	181	172				
		GG	17	33	2.167 (1.093, 4.294)	**0.027**	2.570 (1.123, 5.883)	**0.025**
	Co-dominant	CC	101	58				
		CG	80	114	2.256 (1.470, 3.464)	**<0.001**	2.675 (1.560, 4.586)	**<0.001**
		GG	17	33	3.284 (1.575, 6.847)	**0.002**	3.960 (1.634, 9.600)	**0.002**
	Over dominant	CC+GG	118	91				
		CG	80	114	1.816 (1.222, 2.698)	**0.003**	2.082 (1.271, 3.412)	**0.004**

^1^OR: Unadjusted covariates

^2^OR: Adjusted for educational level, household income, smoking, drinking, exercise duration, frequency of green tea, milk, fruit, spicy food intake, meal times, eating out frequency, hypertension, diabetes, cardiovascular disease and chronic liver disease.

Both univariate conditional logistic regression analysis and multivariate conditional logistic regression analysis for rs1152783 revealed that all five genetic models were statistically significantly associated with ARHL (Table 4).

**Fig 1:**
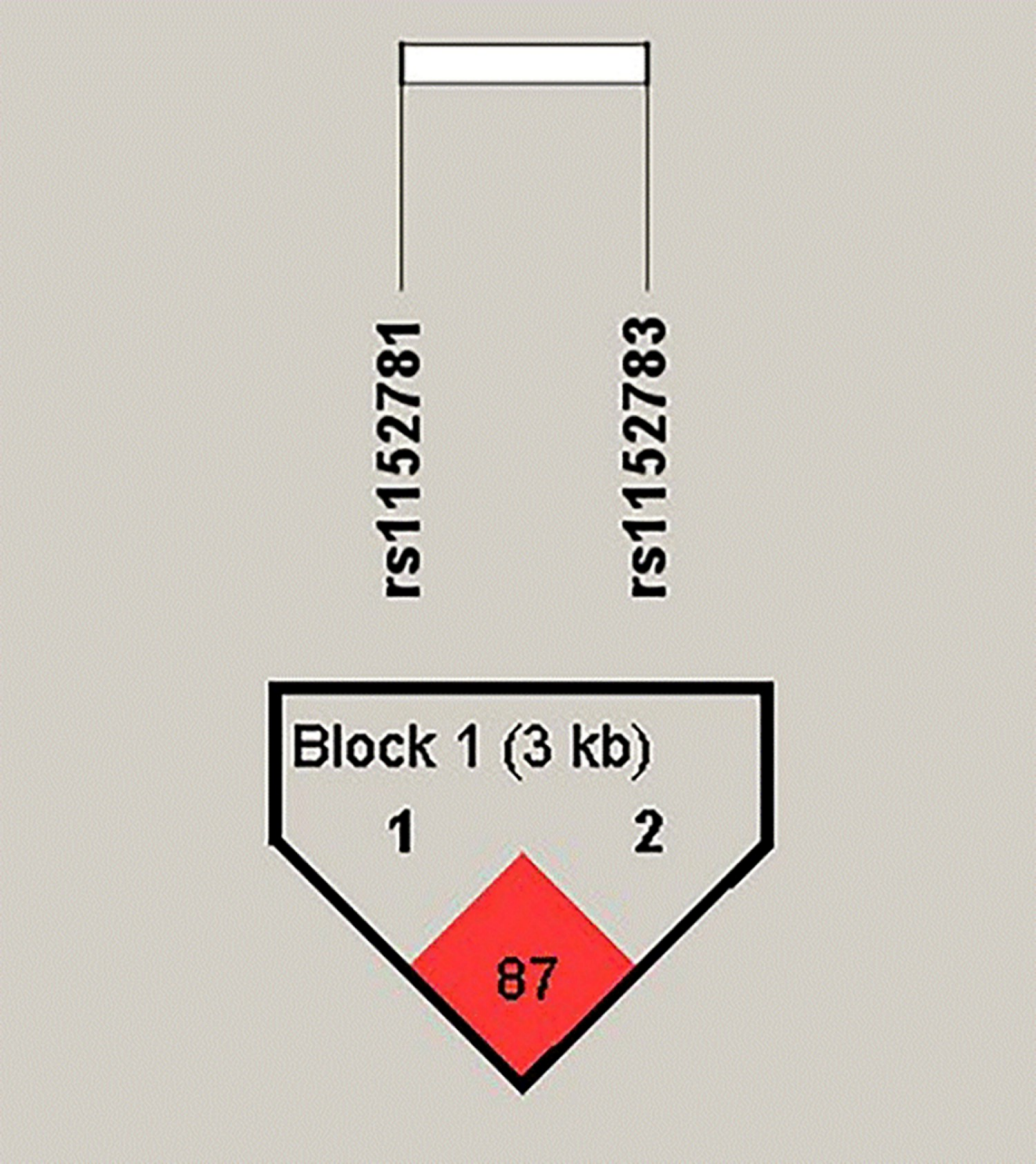
Structure of the haplotype domain of two SNPs of *BCL11B* gene.

The linkage disequilibrium analysis of rs1152781 and rs1152783 of the *BCL11B* gene indicated that the D′ between these two loci was 0.87, which was more than 0.75, r^2^ was 0.118, indicating the presence of linkage disequilibrium (Fig 1). Three haplotypes (TC; TG; GC) were associated with susceptibility to ARHL, the frequency distribution of TC, TG and GC haplotypes showed significant differences between the case group and the control group (*P* < 0.01) (Table 5).

**Table 5:** The haplotype association analysis of *BCL11B* gene.

Haplotype	Haplotype frequency (%)		*χ^2^*	*P*
	Control group	Case group		
rs1152781-rs1152783				
TC	0.560	0.309	52.390	**<0.001**
TG	0.287	0.423	16.578	**<0.001**
GC	0.150	0.252	13.289	**<0.001**

The power (1 − β) for the present study was calculated by applying the formula for the 1:1 matched case-control study and was determined to be 99.99% (> 80%), indicating that the present study yielded statistically significant results.

## Discussion

The association between gene polymorphisms and ARHL has been explored in various researches, and the biological function in various genes expressed in various tissues differed greatly [2,11,12]. We first explored the associations of two SNPs (rs1152781 and rs1152783) of the *BCL11B* gene with ARHL in an older Han Chinese population on the basis of genetic models in the present study. We also constructed haplotype domains for rs1152781 and rs1152783, and we identified three specific haplotypes that were associated with ARHL susceptibility.

In a previous study, an animal experiment revealed that the absence of *BCL11B* expression resulted in the loss of outer hair cells, the impairment of stereocilia, and an increased ABR threshold in mice, ultimately causing experimental mice to suffered from ARHL [17]. However, the mechanism of action by which *BCL11B* affects hearing remains unclear. *BCL11B* is best known for its crucial role in T-cell and neuronal development during embryogenesis [15,25]. However, one study has reported that *BCL11B* is expressed in vascular smooth muscle (VSM) cells and that VSM *BCL11B* regulates aortic stiffness by modulating VSM cytoskeletal actin polymerization via the cGMP (cyclic guanosine monophosphate)/protein kinase G (PKG)/pVASP^S239^ (phosphorylated vasodilator-stimulated phosphoprotein) signaling pathway [26]. Vasodilator-stimulated phosphoprotein (VASP) acts as a key regulator of non-muscle actin polymerization-dependent VSM tone [26,27]. Thus, pVASP^S239^ levels are significantly reduced when *BCL11B* is not expressed [26]. Consequently, these modifications cause non-actin polymerization and an increase in VSM stiffness, which in turn increase aortic stiffness.

Arterial stiffness is a crucial risk factor for adverse cardiovascular outcomes [28–30], which can lead to coronary artery disease (e.g., atherosclerosis) and hypertension [31–34]; atherosclerosis and hypertension are widely recognized as key risk factors for ARHL [35–37]. A possible explanation for this phenomenon is that the variants of SNPs (rs1152781 and rs1152783) alter *BCL11B* expression, thereby further modifying the biological function of *BCL11B* [11]. Given that *BCL11B* may play a major role in the development of ARHL, future research should strive to confirm our findings by conducting additional functional analyses of rs1152781and rs1152783 in the *BCL11B* gene.

In summary, the key novel contribution of this study is the findings regarding the association between *BCL11B* gene polymorphisms and ARHL in a general population. To the best of our knowledge, this study is the first study to report on the potential contribution of *BCL11B* genetic variants to ARHL susceptibility. The statistical power of this study was greater than 80% and the results were reliable, so the main strengths of our study are that the community sample was representative and that the participants in the case and control groups were accurately matched for age and sex to minimize the effects of age and sex-related bias.

Notably, our study had several limitations. Relative to the control group, the case group had fewer participants who had genotypes with homozygous minor alleles of SNPs; this factor could have influenced the stability of our statistical analysis results. To improve the validity of the results, future studies should consider using larger sample sizes. Although our investigators had undergone standardized high-quality training, the effects of information bias on the data-gathering process could not be fully eliminated. Additionally, the results of this study were based on two SNPs of the *BCL11B* gene, which may limit our interpretation of the results and it is necessary to further expand the scope of research.

## Conclusions

The results of this study support the influence of genetic polymorphisms of *BCL11B* on the risk of ARHL in an older Han Chinese population in Qingdao, China. However, further research is required to determine the functional significance of SNPs in relation to ARHL.
